# A Fault-tolerant Steering Prototype for X-rudder Underwater Vehicles

**DOI:** 10.3390/s20071816

**Published:** 2020-03-25

**Authors:** Wenjin Wang, Ying Chen, Yingkai Xia, Guohua Xu, Wei Zhang, Hongming Wu

**Affiliations:** 1 School of Naval Architecture and Ocean Engineering, Huazhong University of Science and Technology, Wuhan 430074, China; d201677414@hust.edu.cn (W.W.); hustxia105@126.com (Y.X.); whm_1995@126.com (H.W.); 2 Department of Mechatronics Engineering, Wuhan Business University, Wuhan 430056, China; erin-ying.chen@outlook.com; 3 Hubei Key Laboratory of Naval Architecture and Ocean Engineering Hydrodynamics, Wuhan 430074, China; 4 Wuhan Second Ship Design and Research Institute, Wuhan 430205, China; dollzw@163.com

**Keywords:** X-rudder AUV, control allocation, fault diagnosis, fault-tolerant control, field tests

## Abstract

The X-rudder concept has been applied to more and more autonomous underwater vehicles (AUVs) in recent years, since it shows better maneuverability and robustness against rudder failure compared to the traditional cruciform rudder. Aiming at the fault-tolerant control of the X-rudder AUV (hereinafter abbreviated as xAUV), a fault-tolerant steering prototype system which can realize dynamics control, autonomous rudder fault detection and fault-tolerant control is presented in this paper. The steering prototype system is deployed on a verification platform, an xAUV, in which the monitor software is developed based on the factory method and the onboard software is developed based on the finite state machine (FSM). Dual-loop increment feedback control (DIFC) is first introduced to obtain smooth virtual rudder commands considering actuator’s limitations. Then the virtual rudder commands are transformed into X-rudder commands based on the mapping theory. In rudder fault diagnosis, an optimized particle filter is proposed for estimating rudder effect deduction, with proposal distribution derived from unscented Kalman filter (UKF). Then the fault type can be determined by analyzing indicators related to the deduction. Fault-tolerant control is addressed by dealing with nonlinear programming (NLP) problem, where minimization of allocation errors and control efforts are set as the optimization objectives, and rudder failure, saturation and actuators limitations are considered as constraints. The fixed-point iteration method is utilized to solve this optimization problem. Many field tests have been conducted in towing tank. The experimental results demonstrate that the proposed steering prototype system is able to detect rudder faults and is robust against rudder failure.

## 1. Introduction

In recent years, a great diversity of autonomous underwater vehicles (AUVs) has been brought into reality, ranging in size from man-portable lightweight ones to large sized vehicles over 10 m in length, with commercial, research, military and other applications. In the pursuit of better maneuverability, X-rudder autonomous underwater vehicles (hereinafter abbreviated as xAUVs) were developed. The first X-rudder underwater vehicle was a research submarine, on which the US Navy carried out numerous maneuver experiments and concluded that the maneuverability is superior to that of all other submarines [1]. Dubbioso, et al. [2] confirmed the superior turning abilities of the X-rudder underwater vehicles by conducting numerical simulations and compared the results to available experimental data. Other scientists [3] have pointed out that xAUVs can still remain operational when the rudder gets stuck, which means the xAUVs have better safety. In addition, xAUVs have better rudder effect, indirectly help improving propulsive efficiency, and are free of damage while docking on the seabed. For these reasons, X-rudders have been adopted more and more in AUV designs around the world. In the civilian field, JAMSTEC (Yokosuka, Japan) has developed a cruising xAUV that can maintain a fixed posture in the horizontal and vertical plane in order to perform exploration in hydrothermal activity areas [4]. MARIN (city, The Netherlands) [5,6] investigated and promoted the capabilities of computational fluid dynamics (CFD) as a tool to conduct 6-DOF free-running maneuvering simulations on a scale physical model equipped with an X-rudder. Although the advantages of the xAUVs are obvious, it is more difficult to control the xAUVs than traditional AUVs, because every control surface of the xAUVs contributes effects on both the horizontal subsystem and the vertical subsystem while in the traditional AUVs they do not. Therefore, control-related problems, such as control allocation, fault detection and fault-tolerant control, should not be ignored and need further research.

Horizontal and vertical planes are used for longitudinal and lateral motion, respectively, in cruciform rudder AUVs. Virtual horizontal and vertical rudder commands are still adopted in the above xAUVs and need to be transformed into X-rudder commands. In [6,7], a PID controller was utilized to produce horizontal and vertical rudder commands first, then a command transformation was employed to calculate the X-rudder commands. However, this command transformation may produce undesired roll torques and affect translational and rotational stability. Aiming at solving this problem, Zhang, et al. [8] formulated the transformation as a constrained minimization problem by introducing the least energy as the criterion, and the pseudoinverse solution of this optimization problem is exactly the transformation formula described in [6,7]. Although the the control allocation problem has been investigated in the existing literature, most research are only conducted using simulations, and there are no field tests in any of the existing literature. Therefore, in this work a fault-tolerant steering prototype system will be designed and an xAUV will be developed as a verification platform. This prototype system is an integrated controller and can realize dynamic control of the xAUV under both normal and rudder failure conditions. In addition, autonomous rudder fault detection will also be included.

Many efforts have been made to the fault detection of AUVs. In order to identify thruster failures, Fagogenis, et al. [9] proposed an algorithm using a mixture of Gaussians and variational Bayes approximations in the diagnosis process, while [10] applied an online Bayesian nonparametric topic modeling technique to characterize the AUV’s performance patterns automatically, then realized fault detection and diagnosis by means of a nearest-neighbor classifier. Reference [11] utilized a second-order sliding mode observer to estimate both the unmeasured system states and the fault extent related to the thrusters of a remotely operated vehicle. In [12], fault diagnosis of the actuators under winding faults was addressed by finite impulse response and principal component analysis. However, most of the above studies were conducted aiming at thruster failures, which may not work for rudder fault detection of the xAUVs. To realize rudder fault detection, a particle filter will be modified with unscented Kalman filter (UKF) for rudder effect estimation.

Fault-tolerant control is another problem in the proposed steering prototype. Wang, et al. [13] implemented fault-tolerant control of underwater vehicles by treating the unknown thruster fault as a part of the general uncertainty, which was tackled by adaptive neural network-based backstepping control. In [14], an adaptive sliding mode observer-based fault-tolerant control design was developed and the fault-tolerant control law was deduced using integral sliding mode technique based on the modified observer and compensation method. Actuator faults were treated as nonlinear disturbances in [15], and the disturbance was estimated by a non-linear disturbance observer. A disturbance observer-based adaptive neural fault-tolerant control scheme was then developed to track the desired system output. In all the above research, the negative effects due to actuator faults were treated as uncertainties or disturbances, then a compensation method was utilized to achieve fault-tolerant control, which greatly increased the complexity of the dynamics controller. To solve this problem, in this paper fault-tolerant control will be decoupled from the dynamics controller design and addressed by nonlinear programming (NLP) in the control allocation process, which releases the complexity of designing the dynamics controller.

Motivated by the aforementioned considerations, a fault-tolerant steering prototype system will be developed in this paper, where a particle filter will be improved for fault detection and NLP will be introduced for fault-tolerant control. The main contributions of this work are summarized as follows:

(1) In order to validate the fault-tolerant steering prototype system, an xAUV will be developed for experimental tests. With modularity and expansibility taken into consideration, monitor software is designed based on factory method, while onboard software is based on finite state machine (FSM). Compared to the existing research, the proposed steering prototype system is unprecedented for xAUVs because of the integration of dynamics control, fault detection and fault-tolerant control. Besides, field tests are conducted firstly for validation.
(2) As for rudder faults detection, the standard particle filter is modified to estimate rudder effect deduction due to faults, where UKF is adopted for providing proposal distribution. Based on the estimation, fault detection can be achieved by analyzing related indicators. To the best of the authors’ knowledge, there is no previous literature about rudder fault detection of the xAUVs.
(3) In the case of rudder failure, fault-tolerant control of xAUVs is addressed by solving multi-objective optimization in this paper. In this optimization problem, minimization of allocation errors and control efforts are considered as optimization objectives, whereas saturation, actuators limitations, and rudder faults are considered as constraints. Compared to previous literatures, this problem is addressed in the control allocation process, rather than in the design of dynamics controller dealing with disturbance.

The remainder of this paper is organized as follows: Section 2 presents the development of the xAUV in terms of hardware composing and software design pattern. Section 3 proposes the scheme of the fault-tolerant control algorithm and presents a detailed process of fault detection. Section 4 presents the simulations of DIFC comparing to other common dynamics controllers. Section 5 validates the previous analysis and design through field tests in a towing tank. Section 6 concludes the work in this paper.

## 2. Vehicle Platform Development

### 2.1. Hardware Components

**Figure 1:**
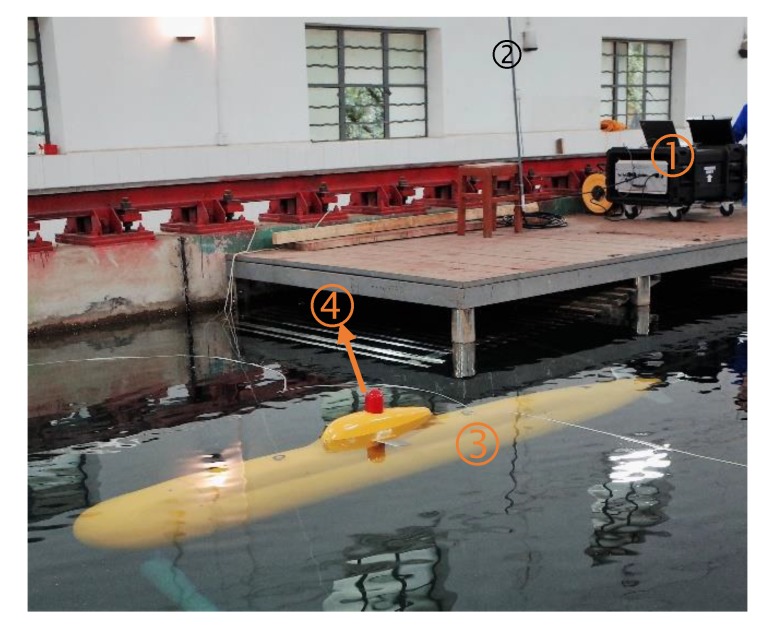
The xAUV sailing in the towing tank. (**1**) Portable surface monitor; (**2**) Surface WIFI antenna; (**3**) xAUV; (**4**) Integrated WIFI&GPS antenna.

The developed xAUV in this work is a scaled physical model. From the point of view of operation, this xAUV is designed to work autonomously or under remote control. As illustrated in Figure 1, an operator can control the xAUV remotely to complete many maneuvers by WIFI integrated into the portable surface monitor. When underwater voyages are required, the operator needs to assign the autonomous tasks to the xAUV firstly, and command the xAUV to switch into the autonomous mode next, then the xAUV will dive into the water and carry out the assigned tasks. After underwater tasks completed, it will float up to the surface and stay there waiting for the next command.

**Figure 2:**
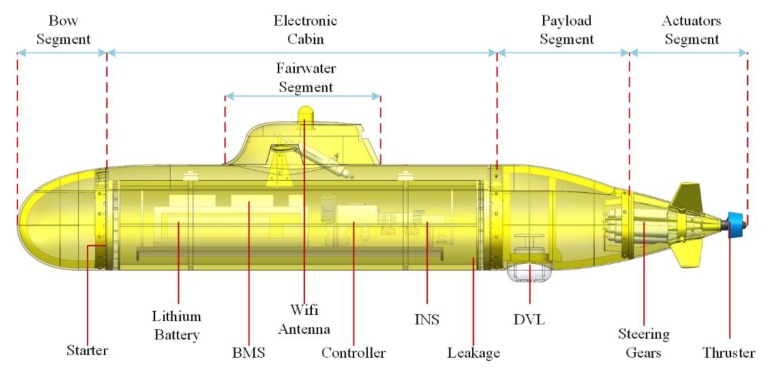
Layout of the xAUV.

The xAUV in this paper is 3 m long and the diameter of the outer contour is 300 mm. A modular construction pattern is introduced in the platform design and the xAUV is divided into five segments: bow segment, electronic cabin, payload segment, actuators segment, and fairwater segment (see Figure 2). There are four electric steering actuators driving the control surfaces, one tunnel thruster in the actuators segment and one steering actuator driving the fairwater rudder in the fairwater segment. This comprises the actuator configuration of the xAUV.

As for the navigation system, an Inertial Navigation System (INS) is utilized to provide position, velocity and attitude with auxiliary data from Doppler velocity log (DVL) and global navigation frame sensor, i.e. GPS. A lithium battery is the only power source for this platform. For safety, a Battery Management System (BMS) is adopted to monitor the health of battery cells, discharging voltage, current, temperature, and state of charge (SOC) of the battery.

**Figure 3:**
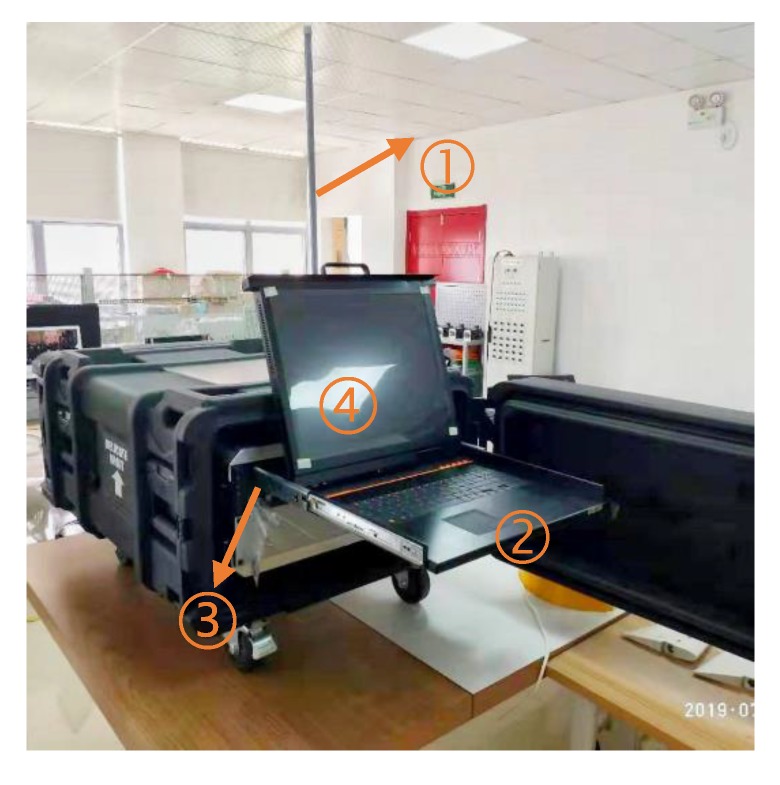
Surface monitor, including (**1**) WIFI antenna; (**2**) KVM; (**3**) Computer; (**4**) Display interface.

The surface monitor is integrated into a portable case along with a computer, a KVM (abbreviation of keyboard, video, mouse) switch and communication instruments (see Figure 3). It can display the status of the xAUV, such as position, velocity, etc. through UDP communication with an onboard computer and send remote commands or task info to the vehicle.

### 2.2. Software Development

#### 2.2.1. Monitor Software Based on Factory Method

**Figure 4:**
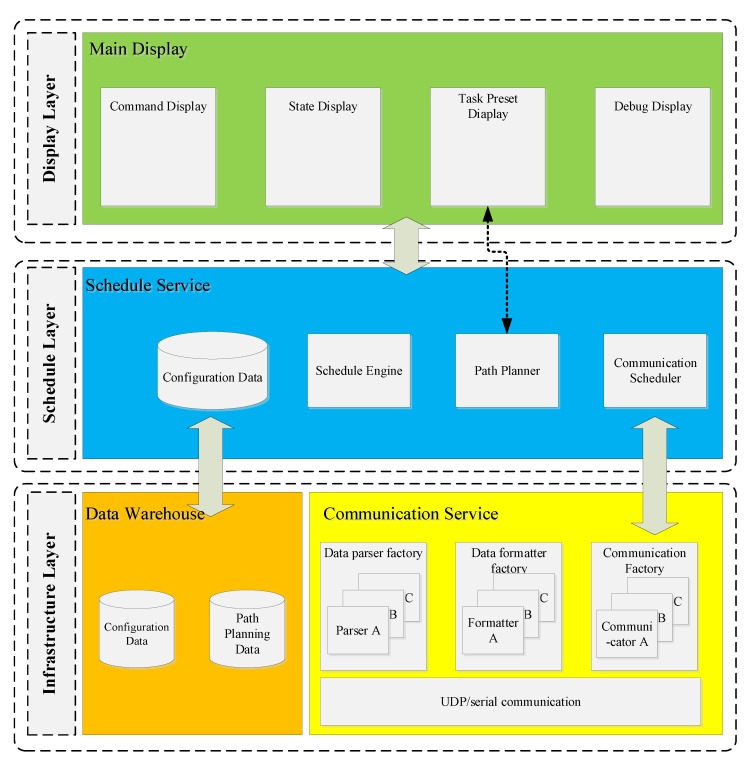
Architecture of factory method-based monitor software.

The monitor software is a supervisory control and data acquisition (SCADA) system [16] and was developed based on the factory method [17], which is a widely used software design pattern concluding the experience in coding, making the code easier to understand, and ensuring the program reliability [18]. As is shown in Figure 4, the monitoring software is developed with three functional layers: display layer, schedule layer, and infrastructure layer. Screens listed in the display layer are designed for human-computer interaction, including state monitoring, sending commands, path planning, etc. The schedule engine in schedule layer takes charge of coordinating the function blocks according to the predefined time sequence and determining the program execution cycle by timers. There are many basic function blocks and data warehouses in infrastructure layer, where a function block can be abstracted into a proprietary factory and a data block can be compared to materials in the factory. With this software construction, the monitor software is highly integrated.

#### 2.2.2. Onboard Software Based on Finite State Machine

In early aquatic vehicle research, the limited hardware capability was a significant challenge for the development of custom software for underwater vehicles [19]. However, with the development of computer science, more and more powerful hardware has become available for researchers and end users. Moreover, open-source middlewares for developing applications for robotic systems come into public eye in recent years. Robot Operating System (ROS) is a popular open-source middleware in the robotics research community because there are already many free function packages and this number is increasing greatly year after year [20]. However, for underwater vehicles, the use of the Mission Oriented Operating Suite (MOOS) maintained by the working group at MIT has been widespread [21]. This is also an open-source middleware that includes many modules specially designed for aquatic vehicles.

**Figure 5:**
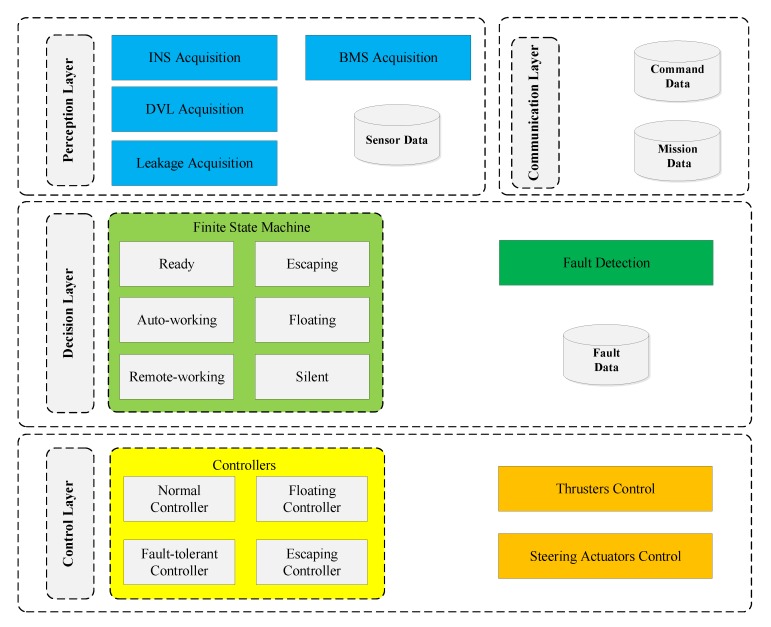
Architecture of FSM-based onboard software.

However, ROS and MOOS are inapplicable for onboard software development of the xAUV, because the controller running Linux is the required platform rather than the Windows CE-based controller from Beckhoff adopted by this xAUV. Therefore, the FSM-based onboard software was customized for the xAUV. FSM is an efficient coding solution for complicated state transitions without nested if-else branches [22] and ensures the xAUV can switch between different working modes correctly and quickly. As shown in Figure 5, the onboard software consists of four layers: perception, communication, decision, and control. Communication layer realizes data exchange with the surface monitor by WIFI. Sensor data is collected in the perception layer and filtered before output. In the decision layer, FSM will determine the working mode of the xAUV considering commands from surface monitor and fault information diagnosed by fault detection block. Motion control algorithms and drive control of the actuators are achieved in the control layer.

**Figure 6:**
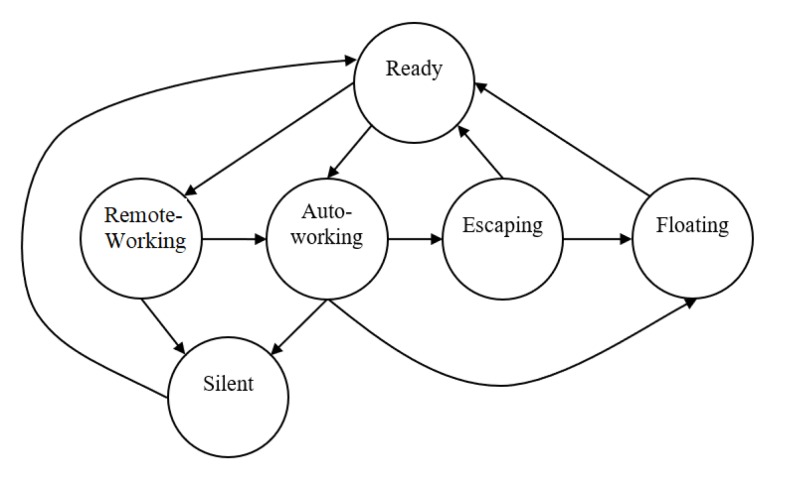
State transition diagram of FSM adopted by onboard software.

The state transition diagram of FSM is shown in Figure 6. Six states for different purposes are designed:

- Ready: Ready to work and feedback states periodically;
- Remote-working: Work under the operator’s commands from the surface monitor;
- Auto-working: Work according to predefined tasks autonomously;
- Escaping: Try to escape if the thruster is twined by ropes or fishing net;
- Floating: Float up to the surface if major failure happens;
- Silent: Keep silent and do not feedback states.

## 3. Fault-Tolerant Steering Algorithms

### 3.1. Steering Under Normal Condition

The xAUV in this paper adjusts its heading or depth usually by X-rudder and the fairwater rudder is used for depth control when necessary. Various control methods for cruciform rudder AUVs have been derived by researchers related to aquatic vehicles. However, there is little literature directly referring to X-rudder control yet. Therefore, it is significant to develop an X-rudder steering method and make good use of its advantages. To achieve this, the four individual control surfaces need to coordinate well since every control surface contributes its own effect to the pitch and heading control. Actually, this manner is quite similar to dynamic positioning systems for ocean surface vessels in [23,24], motion control systems for over-actuated underwater vehicles in [25,26], flight control systems for tailless aircraft with strong interactions between control effectors as described in [27,28].

**Figure 7:**
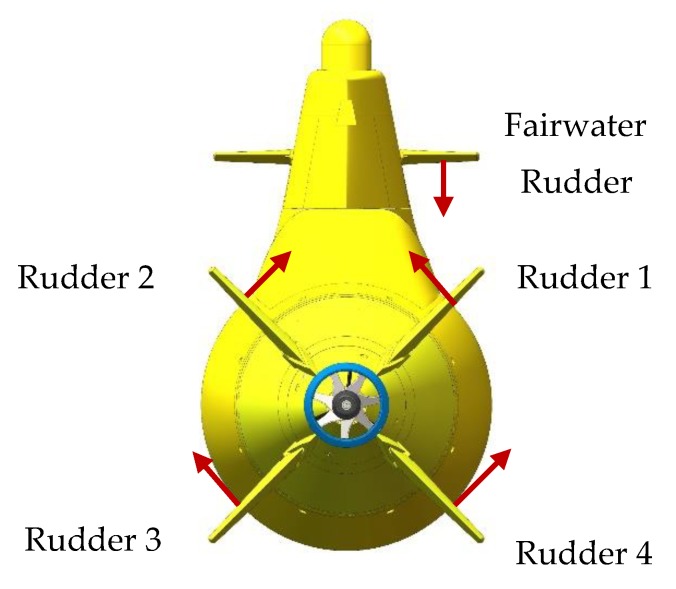
Sign definition of deflection angle. The red arrow represents the positive deflection.

For designing the coordination method, the deflection sign is explained in Figure 7. Upward deflection is defined as positive for stern rudders and the definition of fairwater rudder follows the right-hand rule with the thumb pointing to the starboard side.

#### 3.1.1. Dual-Loop Increment Feedback Control

According to the survey in [29], there is a great diversity of control algorithms for under-actuated or over-actuated underwater vehicles, including PID control, fuzzy control, sliding mode control, adaptive control, backstepping control, optimal control, and many combinations of the above algorithms. These methods have been proved to deal with the uncertainty, environmental disturbances and actuator saturation well that marine vehicles need to consider necessarily. However, this xAUV is designed to sail in a towing tank for debugging in early stage, then carry out some experiments in inland lakes where environmental disturbances, such as winds and currents, are too weak to affect the dynamics. Therefore, it is unnecessary to design a dynamics controller with consideration of disturbance observer [30] and drift compensation [31]. From a practical point of view, the dual-loop increment feedback control (DIFC) is proposed to handle the dynamics control problem and the normal command transformation is developed for control allocation, i.e. the coordination of the four control surfaces.

**Figure 8:**
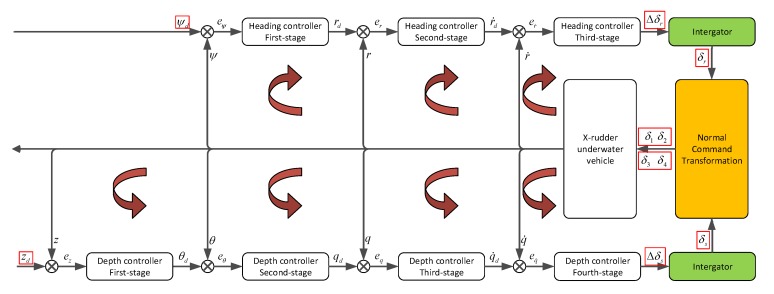
Principle diagram of DIFC. $\delta_{r}$ and $\delta_{s}$ are virtual vertical and horizontal rudder commands respectively. Δδ is the increment, $\delta_{i}\left(i=1,2,3,4\right)$ is the actuator’s control input. $e_{*}$ represents the error. ψ is the heading angle, r is the yaw rate, $\dot{r}$ is the yaw acceleration, z is the depth, θ is the pitch angle, q is the pitch rate, and $\dot{q}$ is the pitch acceleration.

The principle diagram of DIFC is presented in Figure 8. There is a heading controller and a depth controller that each controller has multiple stages forming different control loops. The DIFC will produce the increment of virtual rudder commands $\left(\Delta \delta_{s},\Delta \delta_{r}\right)$, then the integrator sums up the increments and gives out the virtual rudder commands which will be transformed into the actuator’s control inputs $\left(\delta_{i},\;i=1,2,3,4\right)$ by normal command transformation later.

Let $\psi_{d}$ denotes the desired heading, then the heading error $e_{\psi }$, the yaw rate error $e_{r}$ and the yaw acceleration error $e_{\dot{r}}$ are defined as:

(1) $$\left\{ \begin{array}{l} e_{\psi }=\psi_{d}-\psi \\ e_{r}=r_{d}-r\\ e_{\dot{r}}={\dot{r}}_{d}-\dot{r} \end{array}\right.$$

where the heading ψ is usually described as ψ∈[0,2π]. In practice, heading error needs to be modified to deal with heading discontinuity, and the modified expression is presented as:

(2) $$e_{\psi }=\left\{ \begin{array}{ll} \psi_{d}-\psi +2\pi , & when\;\psi_{d}-\psi \in [-2\pi ,-\pi )\\ \psi_{d}-\psi , & when\;\psi_{d}-\psi \in [-\pi ,\pi ]\\ \psi_{d}-\psi -2\pi , & when\;\psi_{d}-\psi \in (\pi ,2\pi ] \end{array}\right.$$

**Figure 9:**
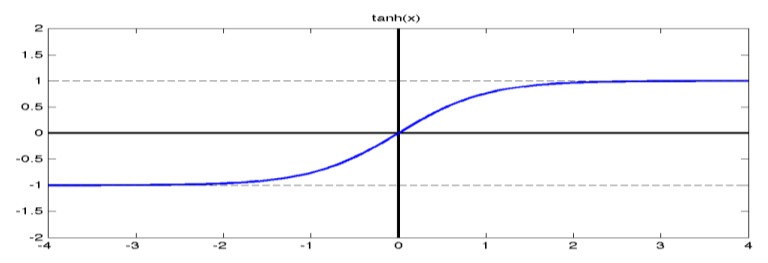
The graphic of the ***tanh*** function.

Based on the above error definitions, the increment heading control law is given as:

(3) $$\left\{ \begin{array}{r} r_{d}=k_{1}\tanh(e_{\psi }/\Delta_{1})\\ {\dot{r}}_{d}=k_{2}\tanh(e_{r}/\Delta_{2})\\ \Delta \delta_{r}=k_{3}\tanh(e_{\dot{r}}/\Delta_{3}) \end{array}\right.$$

where $k_{i}$ is the gain and $\Delta_{i}$ (i=1,2,3) is the scale factor in every loop. $k_{i}$ should be determined according to the characteristics of the xAUV, i.e. $k_{1}$ is determined by max yaw rate, $k_{2}$ is determined by max yaw acceleration, and $k_{3}$ is determined by max rudder deflection speed. $\Delta_{i}$ is used to set the critical error that will produce the maximal outputs. In other words, $\Delta_{i}$ has great effect on the response speed of the control system. The ***tanh*** function is a continuous, symmetrical, smooth and saturated function (see Figure 9), and it is introduced to limit the desired yaw rate, the desired yaw acceleration etc. what’s more, the tanh function is helpful to parameter adaptability since the slop becomes greater when the input approaches to zero. This is a usually adopted manner that a higher gain is preferred when the error comes to the origin, and vice versa.

Finally, the virtual vertical rudder command $\delta_{r}^{t}$ at time t can be derived as:

(4) $$\delta_{r}^{t}=\delta_{r}^{t-1}+\Delta \delta_{r}$$

where $\delta_{r}^{t-1}$ is the last virtual command.

By adopting the virtual vertical rudder command derived from Equation (4), the errors defined in Equation (1) are guaranteed to converge to the origin.

**Proof.**  The proof process is illustrated in the following three steps:

*Step 1*: *Proof of the convergence of*$e_{\dot{r}}$. Consider the following Lyapunov function candidate:

(5) $$V_{1}=\frac{1}{2}e_{\dot{r}}^{2}$$

Differentiating Equation (5), utilizing Equation (3) and (4), one can obtain:

(6) $${\dot{V}}_{1}=e_{\dot{r}}{\dot{e}}_{\dot{r}}\approx e_{\dot{r}}\frac{\partial e_{\dot{r}}}{\partial \delta_{vr}}\frac{\partial \delta_{vr}}{\partial t}\approx e_{\dot{r}}\frac{\partial e_{\dot{r}}}{\partial \delta_{vr}}\left(k_{3}\tanh(e_{\dot{r}}/\Delta_{3})\right)$$

Since $\frac{\partial e_{\dot{r}}}{\partial \delta_{vr}}=\frac{\partial \left({\dot{r}}_{d}-\dot{r}\right)}{\partial \delta_{vr}}=-\frac{\partial \dot{r}}{\partial \delta_{vr}}<0$, it is obvious that ${\dot{V}}_{1}\leq 0$. What’s more, ${\dot{V}}_{1}=0$ can only be obtained when $e_{\dot{r}}=0$. Therefore, the convergence of $e_{\dot{r}}$ is proved.

*Step 2*: *Proof of the convergence of*$e_{r}$. Consider the following Lyapunov function candidate:

(7) $$V_{2}=\frac{1}{2}e_{r}^{2}$$

Differentiating Equation (7), and let $\dot{r}=k_{2}\tanh(e_{r}/\Delta_{2})$ since the convergence of $e_{\dot{r}}$ is guaranteed, one can obtain:

(8) $${\dot{V}}_{2}=e_{r}{\dot{e}}_{r}\approx e_{r}\frac{\partial e_{r}}{\partial r}\frac{\partial r}{\partial t}\approx e_{r}\frac{\partial e_{r}}{\partial r}\dot{r}\approx e_{r}\frac{\partial e_{r}}{\partial r}k_{2}\tanh(e_{r}/\Delta_{2})$$

Due to the fact $\frac{\partial e_{r}}{\partial r}=\frac{\partial \left(r_{d}-r\right)}{\partial r}<0$, it is obvious that ${\dot{V}}_{2}\leq 0$. What’s more, ${\dot{V}}_{2}=0$ can only be obtained when $e_{r}=0$. Therefore, the convergence of $e_{r}$ is proved.

*Step 3: Proof of the convergence of*$e_{\psi }$. Consider the following Lyapunov function candidate:

(9) $$V_{3}=\frac{1}{2}e_{\psi }^{2}$$

Differentiating Equation (9), and let $r=k_{1}\tanh(e_{\psi }/\Delta_{1})$ since the convergence of $e_{r}$ is guaranteed, one can obtain:

(10) $${\dot{V}}_{3}=e_{\psi }{\dot{e}}_{\psi }\approx e_{\psi }\frac{\partial e_{\psi }}{\partial \psi }\frac{\partial \psi }{\partial t}\approx e_{\psi }\frac{\partial e_{\psi }}{\partial \psi }r\approx e_{\psi }\frac{\partial e_{\psi }}{\partial \psi }k_{1}\tanh(e_{\psi }/\Delta_{1})$$

Due to the fact $\frac{\partial e_{\psi }}{\partial \psi }=\frac{\partial \left(\psi_{d}-\psi \right)}{\partial \psi }<0$, it is obvious that ${\dot{V}}_{3}\leq 0$. What’s more, ${\dot{V}}_{3}=0$ can only be obtained when $e_{\psi }=0$. Therefore, the convergence of $e_{\psi }$ is proved.

End proof. □

Similarly, let $z_{d}$ denotes the desired depth, then the depth error $e_{z}$, the pitch error $e_{\theta }$, the pitch rate error $e_{q}$ and pitch acceleration error $e_{\dot{q}}$ are defined as:

(11) $$\left\{ \begin{array}{l} e_{z}=z_{d}-z\\ e_{\theta }=\theta_{d}-\theta \\ e_{q}=q_{d}-q\\ e_{\dot{q}}={\dot{q}}_{d}-\dot{q} \end{array}\right.$$

and the increment of vertical rudder is derived as:

(12) $$\left\{ \begin{array}{r} \theta_{d}=k_{4}\tanh(e_{z}/\Delta_{4})\\ q_{d}=k_{5}\tanh(e_{\theta }/\Delta_{5})\\ {\dot{q}}_{d}=k_{6}\tanh(e_{q}/\Delta_{6})\\ \Delta \delta_{s}=k_{7}\tanh(e_{\dot{q}}/\Delta_{7}) \end{array}\right.$$

where $k_{j}$ and $\Delta_{j}$ (j=4,5,6,7) can be chosen in the same way with heading control law. Then, the actual vertical rudder command is given as:

(13) $$\delta_{s}^{t}=\delta_{s}^{t-1}+\Delta \delta_{s}$$

The stability analysis of depth control can refer to the heading control discussion.

**Remark** **1.** *Since the increment feedback control presented in Equation (4) and Equation (13) is utilized, the static error in heading or depth control due to currents or dead zone of steering actuators can also be eliminated though there is no corresponding compensator in the DIFC scheme, because there is the integration process of the rudder increments and it has been proved that the integration can eliminate the static errors.*

#### 3.1.2. Normal Command Transformation

In Section 3.1.1, control laws for heading and depth control are derived. However, the obtained rudder commands $\delta_{r}$ and $\delta_{s}$ are virtual and need to be transformed into X-rudder commands described as $\delta_{i}\left(i=1,2,3,4\right)$. A fixed mapping formula for transformation is designed as:

(14) $$\left\{ \begin{array}{l} \delta_{1}=\delta_{3}=\delta_{r}-\delta_{s}\\ \delta_{2}=\delta_{4}=-\delta_{r}-\delta_{s}\\ \delta_{i}\in [-\kappa ,\kappa ],\;i=1,2,3,4 \end{array}\right.$$

where κ is the maximum rudder deflection and the diagonal rudders are set the same on purpose so that undesirable roll torque can be avoided. Equation (14) is applicable to most cases except that the calculated $\delta_{i}$ is over the limitation, for instance, $\delta_{r}=25^{{}^{\circ}}$ and $\delta_{s}=-10^{{}^{\circ}}$. This problem was addressed by limiting $\delta_{i}$ within normal range regarding control effort deduction in [7]. For a better solution, Equation (14) should be revised as follows:

(15) $$\left\{ \begin{array}{l} \delta_{1}=\delta_{3}=\delta_{r}-\delta_{s}-\varepsilon_{1}+\lambda \varepsilon_{2}-\left(1-\lambda \right)\varepsilon_{2}\\ \delta_{2}=\delta_{4}=-\delta_{r}-\delta_{s}-\varepsilon_{2}+\lambda \varepsilon_{1}-\left(1-\lambda \right)\varepsilon_{1} \end{array}\right.$$

where λ∈[0,1] is a weight factor representing the importance of depth control, and $\varepsilon_{1}$ and $\varepsilon_{2}$ are the deduction fractions that are defined as:

(16) $$\begin{array}{l} \varepsilon_{1}=\left\{ \begin{array}{ll} \delta_{r}-\delta_{s}-\kappa , & \kappa <\delta_{r}-\delta_{s}\\ 0, & -\kappa \leq \delta_{r}-\delta_{s}\leq \kappa \\ \delta_{r}-\delta_{s}+\kappa , & \delta_{r}-\delta_{s}<-\kappa \end{array}\right.\\ \varepsilon_{2}=\left\{ \begin{array}{ll} -\delta_{r}-\delta_{s}-\kappa , & \kappa <-\delta_{r}-\delta_{s}\\ 0, & -\kappa \leq \delta_{r}+\delta_{s}\leq \kappa \\ -\delta_{r}-\delta_{s}+\kappa , & -\delta_{r}-\delta_{s}<-\kappa \end{array}\right. \end{array}$$

Utilizing Equation (15) and Equation (16), virtual rudder commands $\delta_{r}$ and $\delta_{s}$ calculated by DIFC can be transformed into the individual command $\delta_{i}\left(i=1,2,3,4\right)$. Usually, we are supposed to admit a fact that the depth control is prior to heading control for underwater vehicles, thus λ=1 is selected.

### 3.2. Rudder Faults Detection

Rudder faults are inevitable during experimental operations of AUVs. If this happens, AUVs may lose control, hit a submerged mountain or suffer structural damage due to the extreme depth. Therefore, it is meaningful to detect the faults in time and then take effective measures to maintain maneuverability. In terms of the xAUV developed in this paper, the steering actuators are electrical ball screws that can feed back many states by RS485 communication, such as voltage, current, absolute position, etc. Therefore, communication failure and jam can be considered as two common faults. Worse, the control surfaces may get damaged when hitting an obstacle and this is another typical fault. Typical rudder faults are summarized in Table 1.

Communication failure can be easily detected by monitoring the responses after transmitting a request message [32], since a responsive data acquisition method [33] is utilized for communication between the onboard software and steering actuators. In terms of rudder jam, feedback states are useful to diagnose whether rudder jams happen. The feedback absolute position would remain the same and the feedback current would be nearly equal to the rated value if the electrical ball screw got stuck.

Control surface damage is difficult to detect directly due to the lack of observable characteristic indicators. Aiming at this fault detection problem, a model-based diagnosis is proposed in this paper. Let f(t,x,u) represents the xAUV’s dynamics, and h(t,x) represents the sates measured by sensors, then the nonlinear dynamic system can be described as:

(17) $$\left\{ \begin{array}{l} \dot{x}=f(t,x,u)\\ y=h(t,x) \end{array}\right.$$

where f(t,x,u) is the state transition function in relation to the 6DOF dynamic equations, h(t,x) is the measurement function, x=(p,q,r) is the angular accelerations and y is the measurements, $u=(\delta_{dif},\delta_{r},\delta_{s})$ is the virtual rudder commands. According to the dynamic equations of underwater vehicles based on the Newton-Euler method as presented in [34], the detailed expressions of f(t,x,u) and h(t,x) can be derived as:

(18) $$\begin{array}{l} f\left(t,x,u\right)=M^{-1}\left(-C\left(x\right)x-D\left(x\right)x-g\left(s\right)+Ku\right)\\ h\left(t,x\right)=diag\left(1,1,1\right) \end{array}$$

$M=\left(M_{RB}+M_{A}\right)$ denotes the inertial matrix. $M_{RB}=diag\left(I_{x},I_{y},I_{z}\right)$ is the rigid body inertial matrix. $M_{A}=diag\left(-K_{\dot{p}},-M_{\dot{q}},-N_{\dot{r}}\right)$ is the added inertial matrix.

C(x) accounts for the Coriolis and centripetal matrix, which is presented as:

(19) $$C\left(x\right)=C_{RB}\left(x\right)+C_{A}\left(x\right)$$

where:

(20) $$C_{RB}\left(x\right)=\left[\begin{array}{ccc} 0 & I_{z}r & -I_{y}q\\ -I_{z}r & 0 & I_{x}p\\ I_{y}q & -I_{x}p & 0 \end{array}\right]$$

and:

(21) $$C_{A}\left(x\right)=\left[\begin{array}{ccc} 0 & -N_{\dot{r}}r & M_{\dot{q}}q\\ N_{\dot{r}}r & 0 & -K_{\dot{p}}p\\ -M_{\dot{q}}q & -K_{\dot{p}}p & 0 \end{array}\right]$$

$D\left(x\right)=diag\left(K_{p}+K_{p\left|p\right|}\left|p\right|,M_{q}+M_{q\left|q\right|}\left|q\right|,N_{r}+N_{r\left|r\right|}\left|r\right|\right)$ is the hydrodynamic damping matrix including the linear and quadratic drag.

g(s) represents the restoring moments due to gravity and buoyancy and $s={\left[\phi ,\theta ,\psi \right]}^{T}$. The expression of g(s) is given as:

(22) $$g\left(s\right)=\left[\begin{array}{c} y_{B}F_{B}\cos\theta \cos\phi -z_{B}F_{B}\cos\theta \sin\phi \\ -z_{B}F_{B}\sin\theta -x_{B}F_{B}\cos\theta \cos\phi \\ x_{B}F_{B}\cos\theta \sin\phi +y_{B}F_{B}\sin\theta \end{array}\right]$$

K is the control effectiveness matrix, which is expressed as:

(23) $$K=diag\left(K_{\delta_{dif}},M_{\delta_{s}},N_{\delta_{r}}\right)$$

The symbols used above are defined as follows: $I_{x}$, $I_{y}$ and $I_{z}$ are the moments of inertial; $F_{B}$ is the buoyancy; $x_{B}$, $y_{B}$ and $z_{B}$ are the coordinates of the center of the buoyancy in the body-fixed frame; $K_{\dot{p}}$, $M_{\dot{q}}$, $N_{\dot{r}}$, $K_{p}$, $K_{p\left|p\right|}$, $M_{q}$, $M_{q\left|q\right|}$, $N_{r}$, $N_{r\left|r\right|}$ are the hydrodynamic coefficients which can be directly or indirectly obtained in advance by practical experiments.

In order to derive a diagnostic model, the rudder effect deduction Δu due to specific rudder faults is introduced and Equation (17) is discretized for update in real time, then the discrete model at time k is derived as:

(24) $$\left\{ \begin{array}{l} x_{k+1}=f(k,x_{k},u_{k}+\Delta u_{k})\\ y_{k+1}=h(k+1,x_{k+1}) \end{array}\right.$$

In normal conditions, Δu is supposed to be around zero. In order to estimate Δu, the sate variable x needs to be augmented as $\chi =(p,q,r,\Delta \delta_{dif},\Delta \delta_{r},\Delta \delta_{s})$ and a diagnostic model should be derived. Let ${\hat{\chi }}_{k}$ is the estimation $\chi_{k}$ and ${\hat{y}}_{k}$ is the estimation of $y_{k}$, then the diagnostic model is obtained as:

(25) $$\left\{ \begin{array}{l} {\hat{\chi }}_{k+1}=f(k,{\hat{\chi }}_{k},u_{k})+\nu_{k}\\ {\hat{y}}_{k+1}=h(k+1,{\hat{\chi }}_{k+1})+n_{k+1} \end{array}\right.$$

where $\nu_{k}$ is the covariant of process noise and $n_{k}$ is the covariant of measurement noise at time *k*.

The Kalman filter is a common state filter that has been widely used in underwater vehicles [35], but it requires that process noise ν and measurement noise n are Gaussian and independent whereas real systems are not. For nonlinear and non-Gaussian estimation problems, the particle filter is preferred [9].

As described in [36], a standard particle filter is presented as:

(26) $${\hat{\chi }}_{k}^{}=\sum_{i=1}^{n}\omega_{k}^{i}\chi_{k}^{i}$$

where n is the number of total particles, $\left\{\chi_{k}^{i}\right\}∼q(\chi_{k}|y_{1:k})$ is the particle set, $\omega_{k}^{i}$ is the weight factor determined by the posterior probability $p(\chi_{k}^{i}|y_{1:k})$ and $q(\chi_{k}|y_{1:k})$ is the customized proposal distribution to be selected later. Weight factor $\omega_{k}^{i}$ yields:

(27) $$\left\{ \begin{array}{l} {\tilde{\omega }}_{k}^{i}\propto \frac{p(\chi_{k}^{i}|y_{1:k})}{q(\chi_{k}^{i}|y_{1:k})}\propto \frac{p(y_{k}|\chi_{k}^{i})p(\chi_{k}^{i}|y_{1:k-1})}{q(\chi_{k}^{i}|y_{1:k})}\\ \omega_{k}^{i}={\tilde{\omega }}_{k}^{i}/\sum_{i=1}^{N}{\tilde{\omega }}_{k}^{i} \end{array}\right.$$

where $p(y_{k}|\chi_{k}^{i})$ is the likelihood probability similar to measurement noise and $p(\chi_{k}^{i}|y_{1:k-1})$ is the prior probability. Usually, resampling is necessary for standard particle filters to avoid the degeneracy problem [37], but over resampling will result in particle diversity degradation [38]. Therefore, the unscented Kalman filter (UKF) is introduced into the standard particle filter for providing the proposal distribution $q(\chi_{k}|y_{1:k})$.

The detailed process of fault diagnosis is illustrated as follows:

*Step 1: Initialization.*

Set the initial state ${\bar{\chi }}_{0}=(p_{0},q_{0},r_{0},0,0,0)$ and covariance $\sigma_{0}$, then get the particle set $\left\{\chi_{0}^{i}\right\}(i=1,2,\ldots n)$ including n particles from the gaussian distribution $N(\chi ,{\bar{\chi }}_{0},\sigma_{0})$. Covariance of process noise ν and covariance of measurement noise n are set at the same time.

*Step 2: Prediction.*

Update $\left\{\chi_{k}^{i}\right\}(i=1,2,\ldots n)$ using the system dynamics represented by Equation (25), then a prior probability $\left\{p(\chi_{k}^{i}|y_{1:k-1})\right\}(i=1,2,\ldots n)$ is derived. Next, calculate the mean value ${\bar{\chi }}_{k}$ and the covariance $\sigma_{k}$ basing on $\left\{\chi_{k}^{i}\right\}(i=1,2,\ldots N)$.

*Step 3: Correction.*

Generate the proposal distribution $q(\chi_{k}^{i}|y_{1:k})$ using UKF, and calculate the likelihood probability with the following formula:

(28) $$p(\chi_{k}^{i}|y_{k})\approx N((y_{k}-h(\chi_{k}^{i})),0,\nu )$$

Then, normalized weight factor $\omega_{k}^{i}$ can be calculated by Equation (27) and the estimation ${\hat{\chi }}_{k}$ can be obtained by Equation (26).

*Step 4: Diagnosis.*

Let $\Delta \bar{u}$ is the mean value of actual rudder effect deduction Δu and it yields to $\Delta \bar{u}=0$ in normal condition. Otherwise, $\Delta \bar{u}\neq 0$ when faults happened. Let $\Delta \hat{u}$ is the estimation of Δu and its mean value $\Delta \bar{\hat{u}}$ is defined as:

(29) $$\Delta \bar{\hat{u}}=\frac{1}{L_{1}}\sum_{j=1}^{L_{1}}\Delta \hat{u}(k-j)$$

where $L_{1}$ is selected time slice for diagnosis.

Then, the fault indicator $d_{\Delta u}$ at time k is designed as:

(30) $$d_{\Delta u}(k)=\frac{S_{1}(k)}{\bar{P}}-\ln\left(\frac{S_{2}(k)}{\bar{P}}\right)-1$$

where $S_{1}(k)$ and $S_{2}(k)$ are given as:

(31) $$\begin{array}{l} S_{1}(k)=\frac{1}{L_{1}-1}\sum_{j=1}^{L_{1}}{\left[\Delta \hat{u}(k-j)-\Delta \bar{u}\right]}^{2}\\ S_{2}(k)=\frac{1}{L_{1}-1}\sum_{j=1}^{L_{1}}{\left[\Delta \hat{u}(k-j)-\Delta \bar{\hat{u}}(k)\right]}^{2} \end{array}$$

Finally, a fault is supposed to be present if $d_{\Delta u}(k)>\beta$, otherwise, there is no fault. β is a positive constant determined by past experience.

### 3.3. Fault-Tolerant Control Based on Nonlinear Programming

**Figure 10:**
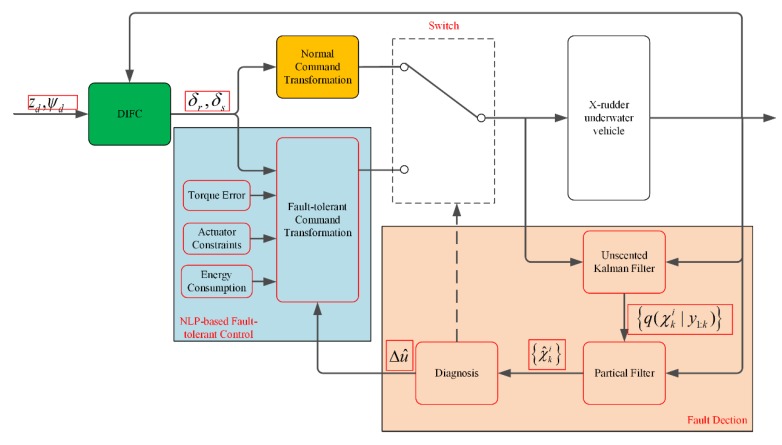
Control block diagram of fault-tolerant steering prototype.

In order to extend the working duration, fault-tolerant control is necessary for the xAUVs to carry out tasks without interruption when rudder failure happens. Thus, a fault-tolerant control scheme including fault detection is presented in Figure 10, where the DIFC, the normal command transformation, the fault detection and the fault-tolerant command transformation are integrated together. In addition, a selector switch is used for selection of transformation methods depending on the rudder health.

Distinguished from traditional fault-tolerant control, modification of dynamics controller is avoided since the fault-tolerant control problem is addressed in command transformation by using the non-linear programming (NLP). Before formulating the programming problem, one can assume that ${\bar{\delta }}_{r}$ and ${\bar{\delta }}_{s}$ are the actual control efforts derived as:

(32) $${\left[\begin{array}{cc} {\bar{\delta }}_{r} & {\bar{\delta }}_{s} \end{array}\right]}^{T}=B{\left[\begin{array}{cccc} \delta_{1} & \delta_{2} & \delta_{3} & \delta_{4} \end{array}\right]}^{T}$$

where the configuration matrix B is given as:

$$B=\frac{1}{4}\left[\begin{array}{cccc} 1 & -1 & 1 & -1\\ -1 & -1 & -1 & -1 \end{array}\right]$$

Then the objective function J of the programming problem can be defined as:

(33) $$J=\left(1-\xi \right)W{\left\|\left(\tau_{d}-\tau \right)\right\|}_{2}+\xi {\left\|u^{t}\right\|}_{2}$$

where minimization of allocation errors and control inputs are considered as criterions. In Equation (33), $\tau_{d}={\left[\begin{array}{cc} \delta_{r} & \delta_{s} \end{array}\right]}^{T}$ is the virtual rudder commands produced by DIFC, $\tau ={\left[\begin{array}{cc} {\bar{\delta }}_{r} & {\bar{\delta }}_{s} \end{array}\right]}^{T}$ is the actual control efforts derived from Equation (32), $u={\left[\begin{array}{cccc} \delta_{1} & \delta_{2} & \delta_{3} & \delta_{4} \end{array}\right]}^{T}$ is the actuator’s control inputs to be solved, *W* is the weight matrix for balancing horizontal and vertical control, and ξ is a parameter for balancing criterion components. When *J* is optimized to its minimum, control allocation error $\left(\tau_{d}-\tau \right)$ is minimum and rudder deflection *u* yields:

(34) $$\left\{ \begin{array}{l} u\in [-\kappa ,\kappa ]\\ \left|\dot{u}\right|\in \left[-\gamma ,\gamma \right]\\ \delta_{i}=D,\;\mathrm{where}i\mathrm{represents}\;\mathrm{the}\;\mathrm{index}\;\mathrm{of}\;\mathrm{faulty}\;\mathrm{rudder} \end{array}\right.$$

where γ is the max deflection rate depending on the actuator’s characteristics, and D is determined according to the fault type and the actuator states. By formulating Equation (34), actuator limitations and rudder faults are considered as constraints.

The optimization problem formulated by Equation (33) and Equation (34) is a common constrained non-linear programming problem and there exist many reliable iterative algorithms [39]. Taking the calculation performance of the onboard system into consideration, the fixed-point iteration method is applied. According to the contraction mapping theorem presented in [40], an iteration formula for calculating the actuator’s control inputs u at time t is designed as:

(35) $$u^{t}=sat\left[(1-\xi )\omega B^{T}W\tau_{d}-(\omega H-I)u^{t-1}\right],\;(where\;\delta_{i}^{t}=\delta_{i}^{t-1}=D)$$

where $H=(1-\xi )B^{T}WB+\xi I^{4\times 4}$, $\omega =1/{\left\|H\right\|}_{2}$, and the definition of sat is given as:

(36) $$sat(\delta )=\left\{ \begin{array}{ll} -\kappa , & \delta <-\kappa \\ \delta , & \delta \in [-\kappa ,\kappa ]\\ \kappa , & \delta >\kappa \end{array}\right.$$

The iteration is terminated when

(37) $$\left\|J(u^{t})-J(u^{t-1})\right\|\leq J_{end}$$

where $J_{end}$ is an adjustable threshold.

By utilizing iteration formula in Equation (35) to solve the NLP presented by Equation (33) and Equation (34), a feasible set of commands for functional rudders can be obtained and its effect can yield to $\delta_{r}$ and $\delta_{s}$ as close as possible. In other words, fault-tolerant control is realized.

**Remark** **2.** *Though the xAUV with rudder faults can maintain the maneuverability by NLP-based fault-tolerant command transformation, its ability to turn or pitch is degraded and limited in fact.*

## 4. Numerical Simulation 

In this subsection, numerical simulations will be conducted to compare the proposed DIFC to PID and Linear Quadratic Regulator (LQR) in terms of accuracy and adaptation to the surge velocity.

In the simulation, the simulated vehicle is required to approach the desired heading and depth under four different surge velocity respectively and the results will be used for performance analysis. During all the simulation scenarios, the initial heading and depth are $\psi_{0}=0$, $z_{0}=0$, and the desired states are $\psi_{d}=90{}^{\circ}$, $z_{d}=5m$. Control parameters of the three control methods are presented in Table 2. For LQR, $G_{1}$ is defined as the input gain, and $G_{2}$ is the feedback gain. For PID, $k_{p}$ is the proportional gain, $k_{i}$ is the integration gain, and $k_{d}$ is the differential gain.

**Figure 11:**
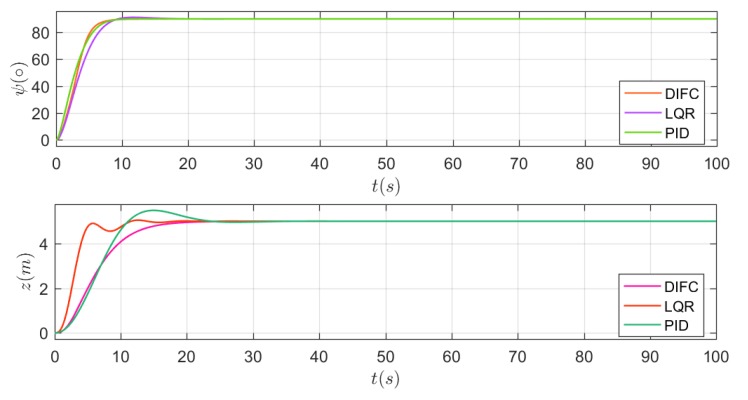
Simulation results when surge velocity is 3 kn. Realtime heading angle ψ during the first scenario (top), and the depth z of the vehicle (bottom).

**Figure 12:**
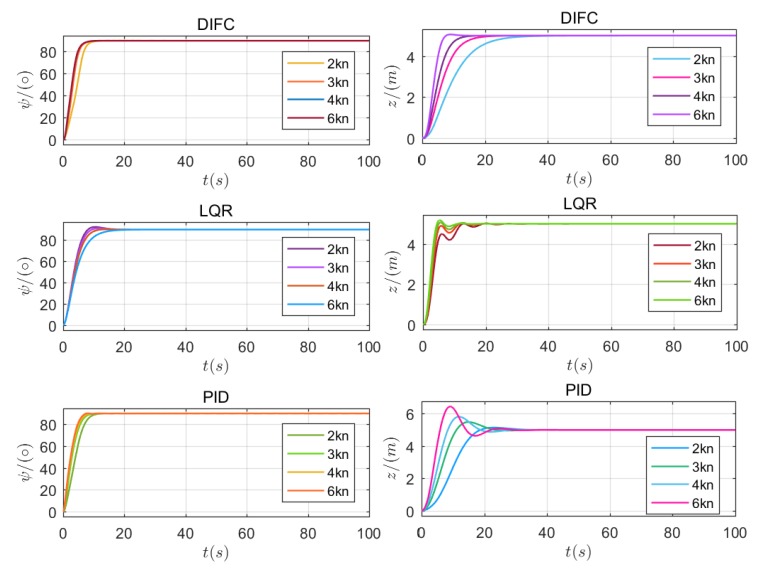
Simulation results under all predefined surge velocity (2 kn, 3 kn, 4 kn, 6 kn). Figures in the first line show the results of DIFC, the second line for LQR, and the third line for PID.

The first scenario contains a heading and depth tracking situation under a cruising speed of 3 kn and the results are shown in Figure 11. The heading control results are nearly the same. The actual course is approaching to 90° gradually and tends to stabilize after 20 s, which indicates that the heading control is satisfactory for DIFC, LQR and PID. However, the depth control results are different. Depth control by DIFC is smoother than with the others, while LQR behaves more rapidly but with slight fluctuations, and PID is accompanied by small overshoot. Therefore, it can be concluded that the DIFC helps to achieve smooth control of heading and depth, which is consistent with the functionality of ***tanh*** function. Simulation results under lower velocity (2 kn) and higher velocity (4 kn, 6 kn) are presented in Figure 12. According to the left three figures, it can be seen that the results under different velocities are nearly the same, which indicates that the surge velocity has little effect on the heading control for any of the methods. Depth control behaves differently under different velocity and this can be found in right three figures. It is obvious that the surge velocity has an effect on depth approaching speed. The slower the velocity is, the slower the depth approaches. In addition, overshoot varies obviously with the velocity in LQR and PID, while there is nearly no overshoot in DIFC and the depth control remain steady all the time. This demonstrates that DIFC has better adaptation to surge velocity comparing to LQR and PID in depth control.

By conducting the simulations, it can be concluded that DIFC can achieve more smooth heading and depth control than LQR and PID, and has better adaptation to surge velocity, which means parameter tuning for different velocity may be avoided.

## 5. Field Tests

### 5.1. Dynamics Control Performance Test

**Figure 13:**
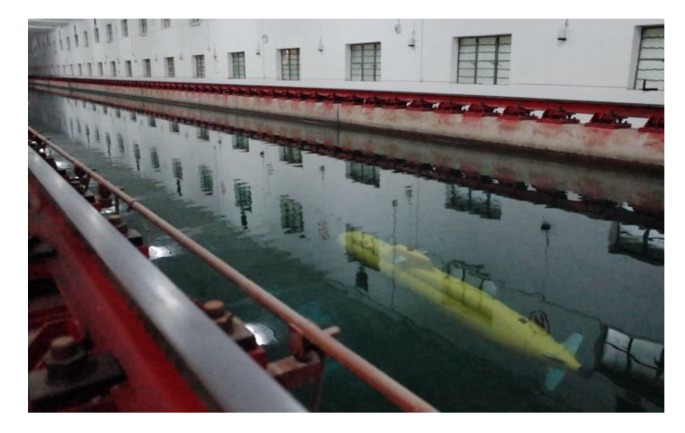
The xAUV is sailing in the towing tank.

In this subsection, the performance of the proposed DIFC is verified by conducting heading and depth keeping trials in a towing tank which is 170 m × 7 m × 4 m (length × width × depth). The assigned task for this xAUV is to keep sailing from west to east at a depth of 2 m. The whole test voyage can be divided into three stages. In the first stage, the xAUV needs to dive with constant stern rudder angles until the depth approaches 1 m for a smooth dive and safety. At the second stage, the DIFC depth controller will be put into use and the xAUV keeps sailing at a depth of 2 m. In the third stage, the xAUV will float up with constant stern rudder angles when the preset time condition is satisfied. During the whole voyage, the DIFC heading controller is operating all the time. The experimental scene is shown in Figure 13, where the xAUV is sailing at 2 m.

**Figure 14:**
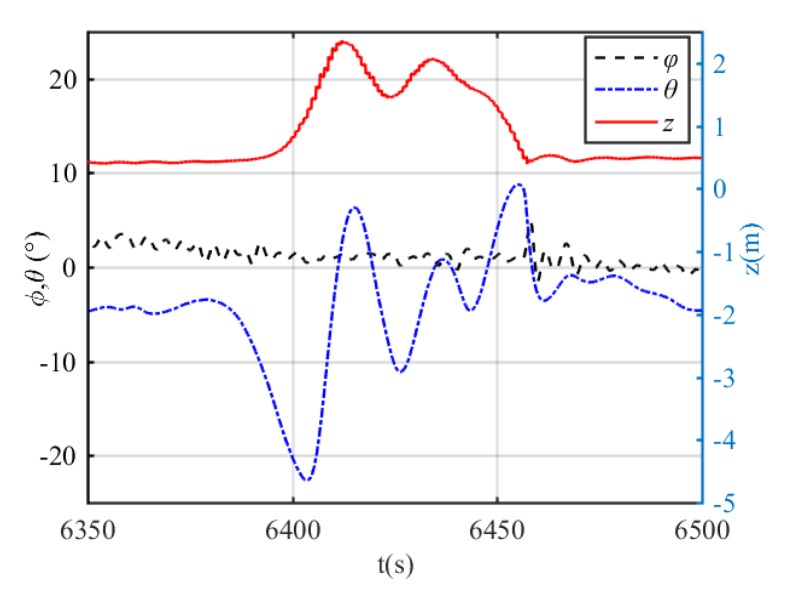
Depth control results. The red line is the depth, the blue line is the pitch angle, and the black line is the roll angle.

**Figure 15:**
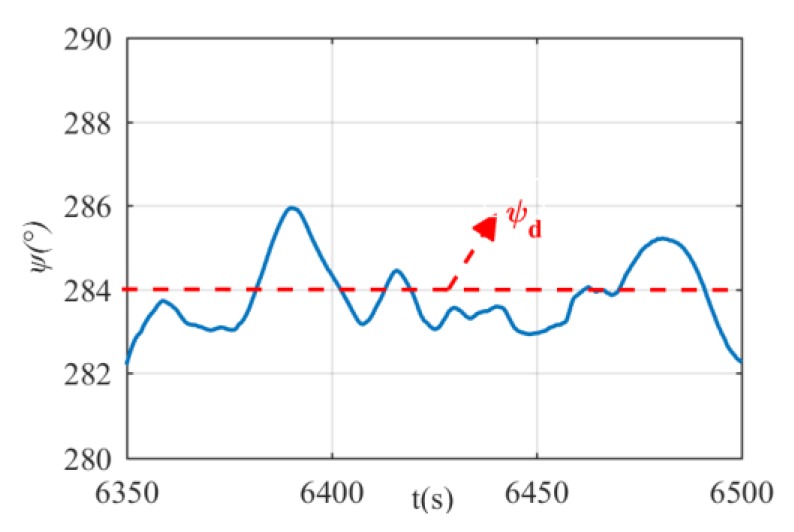
Heading control results. The red line is the heading angle and the red line is the desired heading.

**Figure 16:**
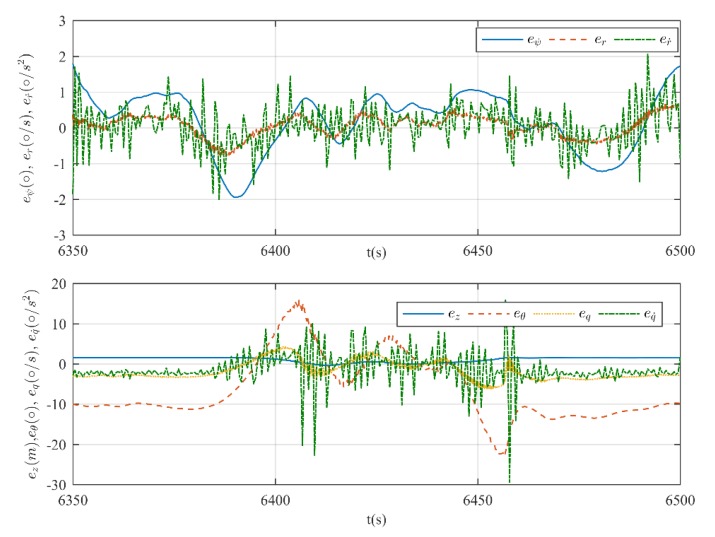
Evolution of errors in every loop. Errors in heading control (top) and errors in depth control (bottom).

The heading and depth tracking results are shown in Figure 14 and Figure 15. It can be seen that the proposed DIFC controller can regulate the xAUV to the desired heading and depth, but there is minor fluctuation in the process. Basing on the results, it is unjust to conclude that the control performance of DIFC is awkward, because the surge velocity is 2 kn and the rudder effect is too weak to stabilize both heading and depth for the xAUV weighed over 235 kg. Therefore, it is reasonable that the xAUV can’t track the desired depth and heading with high accuracy at this relatively low velocity. Unfortunately, tests at higher velocity are prohibited in this towing tank for safety, but according to the simulation results, it should be supposed that a better control performance can be expected if the velocity is increased. Errors in every loop of DIFC are shown in Figure 16. Since the DIFC only operates at the second stage, errors between 6400 s and 6445 s should be used for analysis. In this period, the errors are fluctuating around zero which verifies the convergence of every control loop.

**Figure 17:**
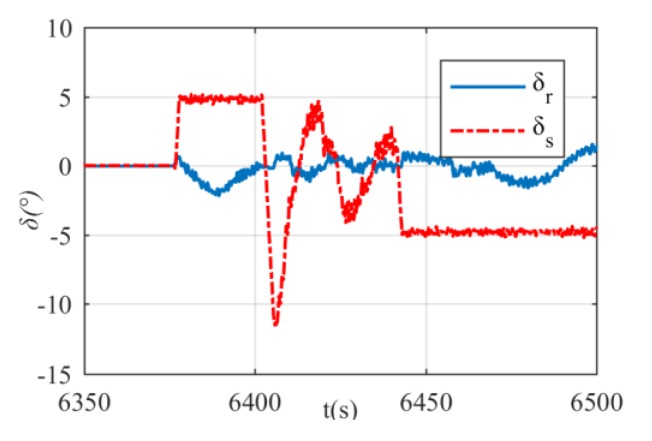
Virtual rudder commands from DIFC. The first stage (6375–6400 s), The second stage (6400–6445 s), The third stage (6445–6500 s).

**Figure 18:**
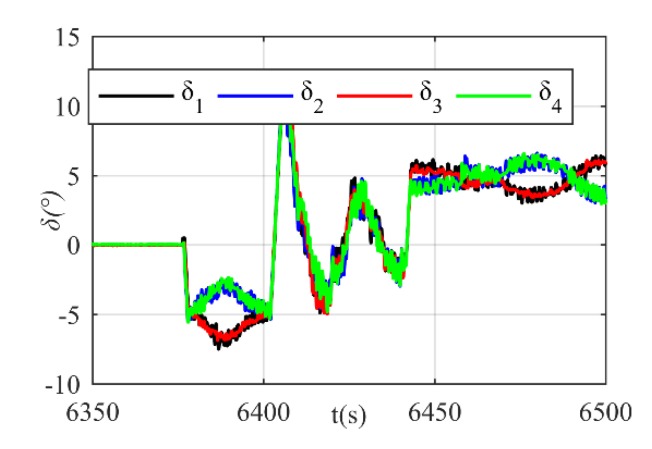
The actuator’s control inputs derived from normal command transformation.

The virtual rudder commands produced by DIFC are shown in Figure 17. Between 6375 s and 6400 s, the xAUV is in the first dive stage and the stern rudder’s deflection $\delta_{s}$ is 5°. Then the xAUV migrates to the second dive stage where depth control by DIFC is activated, and this ends at 6445 s. After that, the xAUV begins to float up with stern rudder deflection of –5° until it comes to the surface. An obvious change of virtual rudder angles can be found only when stage transition occurs. Except that, the variance is a slow-moving thing which reveals the advantage of increment control. The actuator’s control inputs after command transformation are presented in Figure 18. It can be seen that the actuator’s control inputs $\delta_{i}\left(i=1,2,3,4\right)$ are changing with $\delta_{s}$ simultaneously. By further analyzing the command transformation results, it can be obtained that $\delta_{1}$ and $\delta_{3}$ are always the same, so dose $\delta_{2}$ and $\delta_{4}$. Thus, a meaningful find can be obtained that the diagonal rudders are always supposed to deflect in the same direction in normal condition. In this way, the resultant rolling torque can be reduced to its minimum which is necessary for precise heading and depth control, since roll motion will make this control problem more difficult.

### 5.2. Fault Detection Test

**Figure 19:**
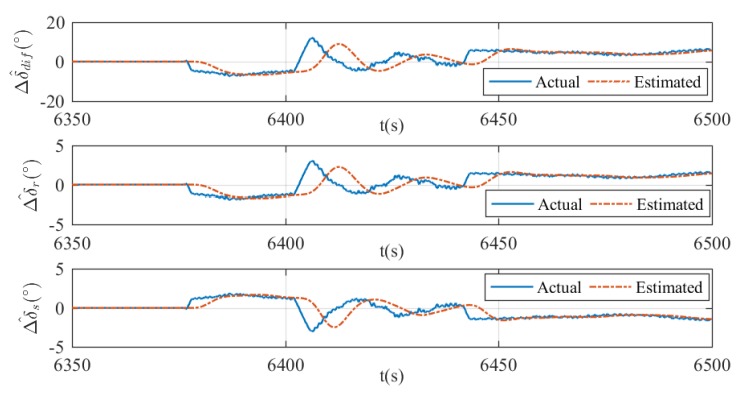
Estimation of Δu in case 1. The estimated differential angle (top), the estimated virtual vertical rudder angle (middle), and the estimated virtual horizontal rudder angle (bottom).

**Figure 20:**
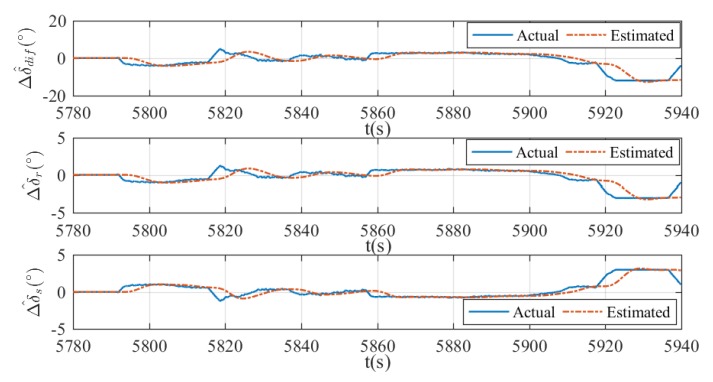
Estimation of Δu in case 2. The estimated differential angle (top), the estimated virtual vertical rudder angle (middle), and the estimated virtual horizontal rudder angle (bottom).

**Figure 21:**
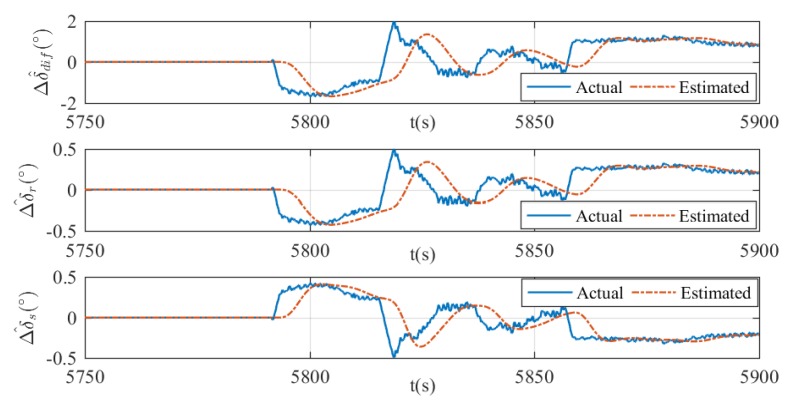
Estimation of Δu in case 3. The estimated differential angle (top), the estimated virtual vertical rudder angle (middle), and the estimated virtual horizontal rudder angle (bottom).

In this subsection, three cases relating to different faults are carried out in order to verify the proposed fault detection method, and the simulated fault is control surface damage which will result in rudder effect deduction. For modelling this fault, the effectiveness factor η is introduced to characterize the fault degree. Since it is improper to destroy the control surface intentionally for conducting the tests, a simulated method is adopted that the real control inputs $\hat{\delta }$ to the actuators are obtained by multiplying the commands $\delta ={\left[\begin{array}{cccc} \delta_{1} & \delta_{2} & \delta_{3} & \delta_{4} \end{array}\right]}^{T}$ calculated by normal command transformation and the effectiveness factor η, but the onboard control system and fault detection program still take δ as the executed commands deliberately. The detailed cases are as follows:

$$\left\{ \begin{array}{l} \mathrm{Case}1:\;\hat{\delta }={\left[\begin{array}{cccc} 0.8 & 1 & 1 & 1 \end{array}\right]}^{T}\circ \delta \\ \mathrm{Case}2:\;\hat{\delta }={\left[\begin{array}{cccc} 0.5 & 1 & 1 & 1 \end{array}\right]}^{T}\circ \delta \\ \mathrm{Case}3:\;\hat{\delta }={\left[\begin{array}{cccc} 0 & 1 & 1 & 1 \end{array}\right]}^{T}\circ \delta \end{array}\right.$$

where ∘ means the Hadamard product. The fault detection results and the estimation of rudder effect deduction $\Delta \hat{u}$ are presented in Figure 19, Figure 20 and Figure 21. In all figures, the moment of fault being triggered can be captured when the actual deduction begins to deviate from zero. In addition, changes of actual deduction mean changes of virtual rudder angles. If the actual deduction keeps constant, one can determine that the virtual rudder angles are also fixed. As is shown in these figures, the estimated deductions are following the actual deductions properly when the virtual rudder angles are changing, for instance, 6375–6450 s in Figure 19, 5890–5920 s in Figure 20, and 5790–5860 s in Figure 21. After the virtual rudder angles stabilize, the actual and estimated deductions approach to the same constant value finally, for example 6450–6500 s in Figure 19, 5920–5939 s in Figure 20, and 5860–5900 s in Figure 21. In summary, the results indicate that the proposed fault detection method can achieve a good estimation of rudder effect deduction with reasonable errors. However, time lag and estimation errors are existent. This is a common phenomenon in the state estimation process and it can be improved by increasing the gains in the filters. In practice, this is a trade-off problem between update rate and system robustness. Usually, the greater the gains are, the faster the update rate is, but the worse the robustness becomes, or vice versa. In terms of this xAUV, slow update rate has no effect on the fault detection results, because the faults are usually irreversible once it happened and it is certain that the faults can be detected finally.

Besides, poor estimation accuracy is also acceptable because a threshold is adopted over which the fault can be determined then. In other words, minor estimation errors won’t reverse the fault detection results. From the point view of robustness, smaller gains are beneficial to decrease the misdiagnoses. Based on the above analysis, it can be concluded that the proposed detection method has acceptable observation ability of rudder effect deduction, thus leading to the accomplishment of diagnosis of rudder faults.

### 5.3. Fault-Tolerant Control Test

Rudder jam is another type of fault which directly affects maneuverability and may bring great danger to the xAUV. In order to verify the robustness of the NLP-based fault-tolerant control proposed in this paper, tests against rudder jam will be carried out in this subsection. The rudder jam settings are listed in Table 3. As for cases 4–6, the tasks assigned to the xAUV are the same as described in Section 5.1.

**Figure 22:**
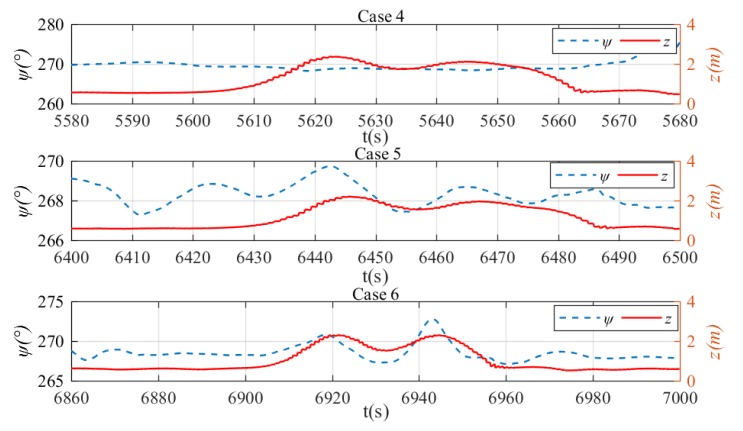
Results of depth and heading control with rudder jam. Heading and depth in case 4 (top), heading and depth in case 5 (middle), and heading and depth in case 6 (bottom).

**Figure 23:**
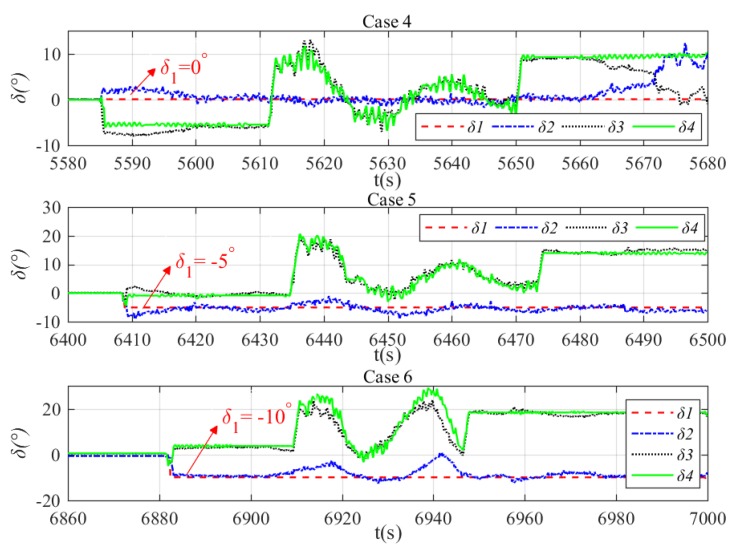
Results of NLP-based command transformation with rudder jam. Rudder commands in case 4 (top), rudder commands in case 5 (middle), and rudder commands in case 6 (bottom).

**Figure 24:**
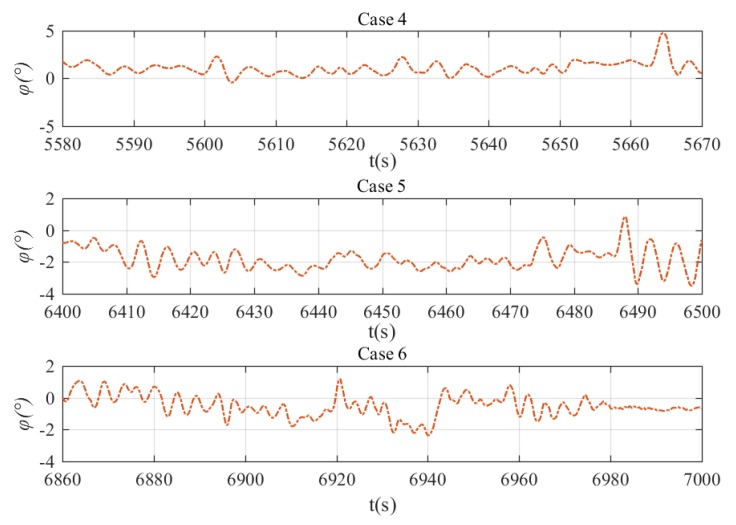
Roll angle during heading and depth control with rudder jam. Roll angle in case 4 (top), roll angle in case 5 (middle), and roll angle in case 6 (bottom).

The depth and heading control results are shown in Figure 22. It can be seen in general that the NLP-based fault-tolerant control method can regulate the xAUV to the desired depth and heading no matter what the stuck angle is. However, there are also oscillations as shown in Section 5.1 and the reasons are the same. What’s more, the oscillations become more violent as the stuck angle increases. In other words, the tracking performance tends to degenerate with the stuck angle increases for the reason that the stuck rudder is likely to produce undesirable roll torque and have negative effects on the control process. Results of NLP-based command transformation are presented in Figure 23. By analyzing the relationship of every rudder’s command, a new rudder transformation pattern can be found in which $\delta_{1}\approx \delta_{2}$ and $\delta_{3}\approx \delta_{4}$, and this is distinguished from the pattern in Section 5.1. After further analyses, the essence of NLP-based fault tolerant control can be found and it can be summarized that $\delta_{2}$ is used for compensation of negative effects produced by $\delta_{1}$, and depth adjustment is achieved by $\delta_{3}$ and $\delta_{4}$ if rudder 1 gets stuck. The roll angle of the xAUV during the tests is presented in Figure 24. It shows that the roll is relatively small and will not make negative effect on the dynamics control. This verifies that the NLP-based rudder transformation method has minimized the negative effect due to rudder jam by coordinating other operational rudders properly. Basing on the above analyses, the proposed NLP-based command transformation has been demonstrated valid in realizing fault-tolerant control with rudder faults.

## 6. Conclusions

This paper addressed the fault-tolerant control problem of X-rudder underwater vehicles under circumstances such as rudder jams, control surface damage, etc. Aiming at solving this problem, a fault-tolerant steering prototype system is designed and its performance is validated by many simulations and field tests. In this prototype system, the monitoring software is developed based on the factory method, while the onboard software is designed based on FSM. Moreover, dynamics control, command transformation, fault detection and fault-tolerant control are all integrated. DIFC is proposed for dynamics control where actuator limitations are considered and smooth virtual rudder commands are obtained. A normal command transformation is designed based on mapping theory to obtain X-rudder commands from virtual rudder commands. The standard particle filter is optimized by proposal distribution derived from UKF and is used to estimate rudder effect deduction. Then the fault can be determined by analyzing the deduction. As to fault-tolerant control, NLP is utilized for fault-tolerant command transformation. Field tests demonstrated that the proposed fault-tolerant steering prototype system is valid and reliable for X-rudder underwater vehicles both in normal condition and rudder failure condition.

However, sensor noise in reality may have negative effects on the performance of dynamic control by DIFC. Thus, the DIFC method will require smooth estimations and its derivatives of those measurements, such as heading angle, pitch angle, etc. so the performance can be guaranteed. As this paper currently focus on the response of the xAUV to the dynamics control under ideal conditions, a study of sensor uncertainties is beyond the scope. An extension of the work to include such filtering method is highly relevant for future work.

Estimations of the rudder effect deduction are highly relevant to the environmental disturbances. In order to obtain a more precise estimation, we have also described a signal separation method based on Fourier analysis that can extract the deduction caused by rudder faults and the effect of disturbances from the original estimations. A detailed analysis is, however, beyond the scope of this paper and remains a topic for future work.

The achievements related to fault detection and fault tolerant control in this study can also be applied to other vehicles, such as over-actuated underwater vehicles, quadrotors, electric vehicles, etc. Since these vehicles also have many independent actuators, fault tolerant control is feasible. In addition, the mathematic models of onshore vehicles are usually more precise than surface vehicles and underwater vehicles. Therefore, it is likely that the accuracy of fault detection implemented on such onshore vehicles are more satisfactory.

## Figures and Tables

**Table 1:** Rudder faults list.

Fault Type	Description
Communication failure	The steering actuator can’t communicate with onboard software normally.
Rudder Jam	Rudder gets stuck at a fixed position and affects maneuverability.
Control surface damage	Control surfaces get damaged due to strike or scratch and rudder effect degraded.
Misalignment	The neutral position changed due to mechanical looseness or structural deformation.

**Table 2:** Control parameters setup.

Method	Subsystem	Parameters
DIFC	Heading Control	$k_{1}=0.38$, $\Delta_{1}=0.5$, $k_{2}=1.2$, $\Delta_{1}=0.06$, $k_{2}=1$, $\Delta_{1}=0.3$
	Depth Control	$k_{4}=0.5$, $\Delta_{4}=3.3$, $k_{5}=0.2$, $\Delta_{5}=0.2$, $k_{6}=1.2$, $\Delta_{6}=0.07$, $k_{7}=1$, $\Delta_{7}=0.3$
LQR	Heading Control	$G_{1}=1.7$, $G_{2}=\left[-1.7,\;-2.1\right]$
	Depth Control	$G_{1}=-1.5$, $G_{2}=\left[1.7,\;-6.9,\;-6.3\right]$
PID	Heading Control	$k_{p}=0.5$, $k_{i}=0.35$, $k_{d}=1.7$
	Depth Control	$k_{p}=10.8$, $k_{i}=1.1$, $k_{d}=2.8$

**Table 3:** Rudder jam settings.

Case no.	Jammed Rudder no.	Stuck Angle
4	Rudder 1	0°
5	Rudder 1	−5°
6	Rudder 1	−10°
